# 免疫检查点抑制剂治疗*EGFR*突变非小细胞肺癌的研究进展

**DOI:** 10.3779/j.issn.1009-3419.2018.08.11

**Published:** 2018-08-20

**Authors:** 日兰 白, 耐飞 陈, 久嵬 崔

**Affiliations:** 130021 长春，吉林大学第一医院肿瘤中心 Cancer Center, the First Hospital of Jilin University, Changchun 130021, China

**Keywords:** 免疫检查点抑制剂, 肺肿瘤, 表皮生长因子受体基因突变, 程序性死亡配体-1, Immune checkpoint inhibitors, Lung neoplasms, *EGFR* mutation, PD-L1

## Abstract

近年来，表皮生长因子受体酪氨酸激酶抑制剂（epidermal growth factor receptor tyrosine kinase inhibitor, EGFR-TKI）已被多项指南推荐作为表皮生长因子受体（epidermal growth factor receptor, *EGFR*）基因敏感突变且不存在耐药的晚期非小细胞肺癌（non-small cell lung cancer, NSCLC）的一线治疗药物，但随着用药时间的延长，大多数患者出现获得性耐药。近几年，针对免疫检查点程序死亡受体（programmed death-1, PD-1）及其配体（PD-1 ligand, PD-L1）的抑制剂取得突破性进展，迅速改变着NSCLC的治疗模式。而近期研究显示，*EGFR*突变NSCLC患者免疫检查点抑制剂疗效尚不理想，可能机制主要包括PD-L1低表达、抑制性免疫微环境及低肿瘤突变负荷等。通过对*EGFR*突变NSCLC患者免疫微环境的变化情况，免疫检查点抑制剂及其与TKI联合应用的研究进展的系列分析，有望为*EGFR*突变NSCLC患者的治疗带来新希望。

## Abstract (en)

In recent years, epidermal growth factor receptor tyrosine kinase inhibitors have been recommended by many guidelines as first-line drugs for advanced non-small cell lung cancer (NSCLC) with *EGFR* gene mutations and no resistance. However, with the prolongation of medication time, most appear acquired resistance. In recent years, breakthroughs in inhibitors of programmed death-1 (PD-1) and its ligand (PD1 ligand, PD-L1) have rapidly changed the therapeutic model of NSCLC. Recent studies have shown that the efficacy of immune checkpoint inhibitors in *EGFR*-mutant NSCLC patients is not satisfactory, which might be caused by low PD-L1 expression, inhibitory immune microenvironment and low tumor mutation load. This review will elaborate the immune microenvironment of NSCLC patients with *EGFR* mutation, the latest study progression of immune checkpoint inhibitors and its combined with TKI, expecting to bring new hopes for the treatment of *EGFR*-mutant NSCLC patients.

表皮生长因子受体（epidermal growth factor receptor, *EGFR*）基因是非小细胞肺癌（non-small cell lung cancer, NSCLC）患者最常见的驱动基因之一^[1]^。近年来，EGFR酪氨酸激酶抑制剂（epidermal growth factor receptor tyrosine kinase inhibitor, EGFR-TKI）已被美国临床肿瘤学会（American Society of Clinical Oncology, ASCO），欧洲肿瘤内科学会（European Society for Medical Oncology, ESMO）和美国国家综合癌症网络（National Comprehensive Cancer Network, NCCN）推荐作为*EGFR*基因敏感突变且不存在耐药的晚期NSCLC一线治疗药物^[2]^，但患者通常在使用此类药物9个月-13个月发生获得性耐药^[3, 4]^，因此亟待开发新的疗法以应对耐药的发生。最近，免疫检查点程序死亡受体（programmed death-1, PD-1）及其配体（PD-1 ligand, PD-L1）抑制剂在肺癌治疗中取得突破性进展^[5, 6]^，但其在*EGFR*突变NSCLC患者，特别是已耐药患者的疗效应答情况尚不清楚。研究者正不断探索其在这一领域的应用，但相关实验治疗结果报道不一。目前的证据显示，*EGFR*突变患者PD-L1表达情况、肿瘤微环境中免疫状态及肿瘤突变负荷等为影响其疗效的主要因素，本文将就这三个方面内容逐一详述。

## 1 *EGFR*突变NSCLC患者PD-L1表达情况

近年来，人们对*EGFR*突变患者PD-L1表达情况进行了多项研究，但结果间存在争议，*EGFR*突变肿瘤细胞PD-L1表达水平为39.5%-71%^[7-10]^，而Gainor等^[11]^评估表达水平仅为11%。一些基础实验显示*EGFR*突变可上调PD-L1表达，从而促进免疫抑制型肿瘤微环境形成。例如，Chen等^[12]^的研究证实表皮生长因子（epidermal growth factor, EGF）的刺激、19外显子缺失和20外显子点突变等可激活*EGFR*基因，随后通过特异性通路（p-ERK1/2/p-c-Jun通路）上调PD-L1表达，诱导T细胞凋亡，而加入到共培养体系中的TKI药物可通过下调PD-L1表达间接提高肿瘤细胞免疫状态。同样，Lin等^[20]^的一项基础研究显示，与EGFR野生型相比，*EGFR*突变细胞系表达更高水平的PD-L1，而用EGF刺激野生型NSCLC后观察到PD-L1表达增加。研究^[13]^还证实，吉非替尼（gefitinib）可通过核因子κB（nuclear factor κB, NF-κB）信号通路下调PD-L1表达，可能成为TKI药物治疗肿瘤患者的机制之一。Zhang等^[14]^证明激活的EGFR基因可通过白细胞介素6/Janus激酶/信号转导和非小细胞肺中转录激活因子3（IL-6/JAK/STAT3）信号通路上调肿瘤细胞PD-L1表达。Akbay等^[7]^在小鼠模型中发现*EGFR*突变细胞系表达PD-L1，并且在使用PD-1/PD-L1抑制剂后肿瘤减小，小鼠存活时间延长，提示免疫治疗在*EGFR*突变肿瘤中可能存在潜在作用。

然而，最近多项临床研究显示*EGFR*突变对PD-1/PD-L1抑制剂反应率较低，对PD-L1表达存在负向调控作用^[11, 15, 16]^。Li等^[15]^对4, 857例肺癌患者数据进行的*meta*分析显示，EGFR野生型与突变型肿瘤PD-L1^+^表达率具有显著差异（44.1% *vs* 36.7%, *P* < 0.05）。Dong等^[16]^对15项研究3, 283例肺癌患者进行的分析也显示出相同结果（*P*=0.02）。为进一步证实，研究人员检测了癌症基因组图谱（The Cancer Genome Atlas, TCGA）和广东肺癌协会（Guangdong Lung Cancer Institute, GLCI）数据库中NSCLC患者肿瘤样本的mRNA谱、PD-L1蛋白免疫组化检测和反相蛋白阵列（reverse phase protein arrays, RPPA），并对255例NSCLC手术标本进行了免疫组化和Sanger测序，同样发现*EGFR*突变组患者PD-L1表达呈现低水平，EGFR野生组PD-L1表达强阳性比例显著高于突变组（*P* < 0.05）^[16]^。最近，Haratani等^[17]^分析了TKI治疗期间疾病进展后用纳武单抗（nivolumab）治疗的患者的PD-L1表达情况及其与T790M状态之间的关系，结果显示T790M阴性患者较T790M阳性更能从PD-1抑制剂治疗中获益（中位PFS分别为2.1个月和1.3个月），而PD-L1水平≥10%或≥50%的肿瘤细胞比例也更高于T790M阳性患者。这一研究为T790M阴性NSCLC耐药患者，特别是同时伴PD-L1阳性表达的患者的免疫治疗决策提供一定帮助。

不同试验研究结果相互矛盾可能与以下因素有关：PD-L1检测技术及样本来源的异质性^[18-22]^；除肿瘤细胞外，TIL上亦可检测出PD-L1的表达^[23]^；此外，使用TKI药物期间及耐药后肿瘤患者免疫状态发生动态改变。Gainor等^[11]^的研究报道了TKI使用前后PD-L1的变化情况，显示TKI治疗前PD-L1表达≥1%、≥5%和≥50%的患者分别占24%、16%和11%，而TKI治疗耐药后分别占31%、29%和14%。28%（16/57）患者在TKI耐药后PD-L1表达水平发生动态改变，其中21%（12/57）表达上调，7%（4/57）表达下调。此外，PD-L1^+^（表达≥5%）与高CD8^+^ TILs浸润（≥2级）同时出现的患者在治疗后也明显增加（11.6%和2.1%）。Han等^[24]^的研究也显示*EGFR*突变NSCLC患者吉非替尼耐药后导致38.9%（7/18）肿瘤细胞中PD-L1表达显著增加。这与Jiang等^[25]^的研究结果一致，在鼠类肉瘤滤过性毒菌致癌性同源体B1（v-raf murine sarcoma viral oncogene homolog B1, *BRAF*）基因抑制剂治疗黑色素瘤耐药后的活检组织中发现PD-L1表达增加，考虑可能与TKI耐药后肿瘤组织发生间质上皮转化（mesenchyal epithelial transition, MET）以及表达PD-L1免疫细胞，例如调节性T细胞（regulatory T cell, Treg）数量的增加有关^[24]^。*EGFR*突变NSCLC患者在使用TKI耐药前后PD-L1表达水平和TIL浸润发生动态变化，部分呈现PD-L1表达和/或TIL浸润显著增加现象，可能解释一些患者耐药后二线免疫治疗疗效较好。

此外，一些试验仅在研究EGFR状态与PD-L1表达之间的相关性，并没有涉及肿瘤突变负荷（tumor mutation burden, TMB），肿瘤微环境中免疫细胞及其他分子的表达分析。因此，综合考虑*EGFR*突变患者免疫状态，并动态监测耐药后的免疫微环境变化对下步治疗决策至关重要。

## 2 *EGFR*突变影响肿瘤微环境免疫状态

### 2.1 *EGFR*突变影响CD8^+^ T细胞

肿瘤微环境中浸润的淋巴细胞和炎性细胞已证实与NSCLC患者生存率相关^[26, 27]^，可能成为预测免疫治疗效果的重要生物标志物^[28]^。多项研究显示*EGFR*突变基因与肿瘤微环境中TIL低度浸润相关。Mazzaschi等^[29]^研究发现*EGFR*突变者CD8^+^和PD-1^+^ TIL细胞在肿瘤内的密度显著减少。Dong等^[16]^通过汇聚GLCI数据库中mRNA表达情况证实*EGFR*突变型肿瘤CD8^+^ TIL明显低于野生型患者（*P*=0.031），随后在255例NSCLC手术标本中也发现相同结果（*P*=0.003）。不仅如此，对GLCI数据库PD-L1和CD8^+^ TIL进行的联合检测显示，与EGFR野生组相比，*EGFR*突变体组双阴性（PD-L1^-^/CD8^-^ TIL）比例显著高于双阳性（PD-L1^+^/CD8^+^ TIL）比例（*P* < 0.001）；同样，双阳性组中*EGFR*突变情况呈低水平，双阴性组*EGFR*突变呈显著高水平（*P*=0.005）^[16]^，表明*EGFR*突变患者肿瘤微环境可能为非炎症型。Gainor等^[11]^的回顾性研究也证实了这一点：*EGFR*突变患者PD-L1表达量及T细胞浸润程度均较低，且几乎没有CD8^+^ TIL浸润和PD-L1高表达共同出现的病例。Haratani等^[17]^的结果显示，*EGFR*突变患者中对纳武单抗有反应者体内CD8^+^ TIL密度更高；另外，T790M阳性组和阴性组患者的CD8^+^ TILs数量相似，但T790M阴性组PD-L1高表达（≥10%）和高CD8^+^ TIL浸润（≥中值）共存现象多于阳性组[20%（5/25）和4%（1/24）；12%（3/25）和4%（1/24）]，T790M阴性组患者的FOXP3^+^ TIL计数显著低于阳性组，提示免疫治疗可能是T790M阴性者的潜在二线选择。

### 2.2 *EGFR*突变影响Treg细胞

Treg是一类对自身免疫反应起负调控作用的细胞群，可以分泌白介素-10（interleukin-10, IL-10）和转化生长因子-β（transforming growth factor β, TGFβ）帮助肿瘤产生免疫抑制环境，减弱CD4^+^ T细胞、CD8^+^ T细胞及NK细胞产生的抗肿瘤效应^[30]^，所以肿瘤患者Treg细胞的数量通常会明显增加^[31, 32]^。Huang等^[33]^的研究证实，肿瘤中*EGFR*突变基因可上调Treg细胞的生成，下调肿瘤特异性杀伤细胞的表达，从而影响抗肿瘤疗效。研究^[34]^表明，核内转录因子Foxp3是Treg细胞功能性的特异性标志。Wang等^[35]^研究发现，糖原合酶激酶-3β（glycogen synthase kinase 3β, GSK-3β）可直接或通过β-连环蛋白（β-catenin）和白细胞淋巴瘤/白血病xL（Bcl-xL）的表达间接下调Foxp3蛋白的表达，进而抑制Treg细胞功能。而在多种肿瘤细胞中表达均上调的双调蛋白（amphiregulin, AREG）^[39]^，可通过EGFR信号下调GSK-3β蛋白的活性，促进Foxp3蛋白翻译后的修饰过程，进而维持Treg细胞的抑制功能。Haratani等^[17]^发现T790M阴性患者的Foxp3^+^ Treg细胞计数显著低于阳性患者。在肺鳞癌的小鼠模型研究中^[36, 37]^，观察到敲除*EGFR*基因后肿瘤微环境中浸润的Foxp3^+^ Treg细胞较少。因此*EGFR*突变可以改变肿瘤微环境Treg细胞免疫状态，促进肺癌的生长。

### 2.3 *EGFR*突变影响肿瘤微环境其他因素

由肿瘤特异的T细胞产生的干扰素-γ（interferon-γ, IFN-γ）可识别肿瘤细胞或抗原递呈细胞上相应受体、招募其他免疫细胞或直接抑制肿瘤细胞增殖，从而发挥有效的抗肿瘤免疫效应^[38]^。Chen等^[12]^的研究显示使用TKIs后可提高IFN-γ的产生，证实*EGFR*突变对肿瘤微环境中IFN-γ有一定的抑制作用。CD73可促进ATP生成腺苷，通过腺苷旁路上调Treg细胞的表达，改变肿瘤细胞和免疫细胞功能，使机体呈现免疫抑制微环境。同时，CD73的表达可被EGF诱导，应用TKIs后CD73也会减少。2017年ASCO报道的一项研究探索分析了TCGA和CP1108数据库，发现EGFR突变NSCLC患者PD-1/PD-L1抑制剂疗效较差。研究者对患者标本mRNA测序结果显示此类患者存在CD73高表达和IFN-γ表达下调现象，揭示了其免疫抑制治疗效果不佳的相关机制。研究者还提出了将CD73抑制剂与TKIs或PD-1/PD-L1抑制剂联合使用的可能性，为靶向治疗耐药患者的治疗提供了新思路。

不论是CD8^+^ TIL细胞和IFN-γ的下调，还是Treg细胞和CD73表达的上调，都表明*EGFR*突变基因对NSCLC患者肿瘤微环境免疫状态有重要影响，非炎症型免疫微环境是此类患者肿瘤发生的重要因素，也是其无法从免疫治疗中获益的部分原因。针对这些因素改善肿瘤患者微环境免疫状态或可成为该类患者的治疗途径，如抑制Treg细胞、CD73抑制剂或过继CD8^+^ TIL细胞后再联合免疫抑制剂等。

## 3 *EGFR*突变影响肿瘤突变负荷

肿瘤基因组的突变是一个动态连续的过程，包括DNA修复酶、抑癌基因失活及癌基因激活等，促进肿瘤发生、复发和转移^[39]^。最近试验^[40]^显示TMB是免疫抑制剂疗效预测的良好生物标志，可定量估计肿瘤基因组编码区的突变总数。研究表明TMB越高，肿瘤产生的新抗原越多，越易被免疫细胞识别，因此对免疫治疗有更强的应答。单个点突变如*EGFR*、*BRAF*和*TP53*突变通常是NSCLC早期克隆驱动事件，目前研究显示这类特定基因突变患者多数对免疫治疗反应不理想，而减少的TMB可能是另一种重要的影响因素。

Haratani等^[17]^为研究*EGFR*突变NSCLC耐药后使用纳武单抗治疗疗效与患者TMB之间的关系，对队列A中9例患者（包括3例对治疗有反应者和6例无反应者）进行了全外显子组测序，结果显示每个肿瘤患者的中位TMB为101，且对药物有应答的患者的TMB显著高于无反应患者，但T790M突变阴性与阳性患者之间没明显差异。同样，Spigel的研究^[41]^也显示*EGFR*突变患者具有低TMB。由此推测，部分患者PD-L1高表达和TIL高浸润但免疫抑制剂反应差可能与低TMB导致的低肿瘤新抗原密切相关，随后TMB在*EGFR*突变NSCLC免疫抑制剂反应中的影响不断引起了研究者的关注。Dong等^[16]^分析了TCGA和Broad数据库的患者，发现TCGA数据库中*EGFR*突变型（外显子19Del、L858R、L861Q、G719X、S768I；突变负荷中位值为56）与野生型TMB（突变负荷中位值为181）间存在明显差异；Broad数据库显示EGFR野生型突变负荷中位数明显高于突变型（209:59）。为证实这一观点，研究人员接着对GLCI数据库的85个肺腺癌进行全基因组测序，仍得出相同结论（*EGFR*基因野生型与突变型的突变负荷中值比为197:162）。

目前关于*EGFR*突变与TMB的相关性研究还在进行中，而微卫星高度不稳定（microsatellite instability-high, MSI-H）、错配修复基因缺失（mismatch-repair deficiency, dMMR）在免疫治疗中的作用也是未来研究中需要不断探索的部分。此外，免疫疗效预测其他潜在的生物标志物，如T细胞库克隆性、外周血细胞因子和多重免疫组化（multiplex immunochemistry）等尚需在*EGFR*突变NSCLC患者中进行深入探索。

## 4 免疫检查点抑制剂及联合应用治疗*EGFR*突变NSCLC患者的研究进展

### 4.1 免疫检查点抑制剂的研究进展

*EGFR*突变基因可影响NSCLC免疫检查点抑制剂的疗效反应，基于上述*EGFR*突变基因造成的PD-L1低表达、抑制性免疫微环境及低TMB等的免疫现状，最近的多项临床试验显示，此类患者无法从免疫治疗中获益，甚至疗效更差。CheckMate057试验^[42]^和KEYNOTE010试验^[43]^对二线PD-1/PD-L1抑制剂与多西他赛（docetaxel）治疗NSCLC的疗效进行了对比，结果显示，具有*EGFR*突变的患者在总生存期（overall survival, OS）上无法从PD-1/PD-L1抑制剂中获益，在无疾病进展时间（progression free survival, PFS）上甚至差于多西他赛，而EGFR野生型患者在OS和PFS上均显示明显受益。最近发表的一项纳入28例PD-1/PD-L1抑制剂治疗晚期肺腺癌的回顾性分析^[44]^显示，伴*EGFR*突变的患者客观缓解率（objective response rate, ORR）仅为5%。Gainor等^[11]^在纳入58例患者的回顾性分析中，重点关注了使用PD-1/PD-L1抑制剂的*EGFR*突变患者，发现与EGFR野生组相比，突变组患者的PFS和ORR显著降低。对疗效的研究不仅可从纳入多项病例的临床试验的综合分析出发，还可以在单个病例报道中得到启示。例如，Dong等^[16]^试验介绍了用派姆单抗（pembrolizumab）治疗*EGFR*突变NSCLC患者的典型病例。1例是*EGFR*-L858R突变肺腺癌患者，重度吸烟，PD-L1阴性，CD8^+^ TIL呈低密度，经治疗2个周期后病情急剧进展；另1例是*EGFR*基因19外显子缺失的肺鳞癌患者，不吸烟，PD-L1强阳性，CD8^+^ TIL呈高密度，在治疗3个周期后，该患者几乎没获益且疾病很快进展。这些临床试验和单个病例报道均显示*EGFR*突变NSCLC患者的免疫疗效远不如之前报道的那么乐观。

然而，Garassino等^[45]^进行的ATLANTIC研究显示，PD-L1表达超过25%、伴有EGFR^+^/ALK^+^的患者，也可从免疫治疗中获益。该研究对2014年-2015年期间444例局部晚期不可手术的Ⅲ期NSCLC患者进行了PD-L1抑制剂（durvalumab）治疗，结果显示与安慰剂相比，durvalumab可延长患者PFS超过11个月。但意外的是，对于EGFR^+^/ALK^+^且PD-L1表达阳性的NSCLC组，三线使用durvalumab的治疗应答率可达12.2%（9/74），并且其安全性与其他PD-1/PD-L1抑制剂一致。该研究还显示，在使用durvalumab 21个月后，PD-L1表达超过25%的EGFR^+^/ALK^+^患者中，之前疗效应答者有56%（5/9）仍为无进展生存，而PD-L1表达低于25%者在获得PR后疾病均发生进展^[40]^。这提示，根据PD-L1表达情况可能在免疫治疗前选择可受益的适合人群，成为*EGFR*突变患者进行免疫治疗疗效预测的生物标志物，但仍需进一步探索以确认PD-L1阈值以及与其他标志物的联合应用。尽管如此，该研究仍有几点值得注意。首先，实验并没有设立独立对照组，组间实验患者的差异（如人口统计学和疾病特征）可能影响实验结果，因此应进一步对EGFR^+^/ALK^+^亚组进行独立对照分析；其次，各队列随访持续时间不一致，需对后续研究数据进行分析，以明确EGFR^+^/ALK^+^与EGFR^-^/ALK^-^ NSCLC组反应持续时间是否相似；最后，该研究并没有分析durvalumab治疗EGFR^+^/ALK^+^患者的疗效与TMB的相关性，以及EGFR^+^患者疾病进展与T790M的相关性^[45]^。

*EGFR*突变NSCLC患者对免疫检查点抑制剂的疗效反应的诸多研究结果存在矛盾，虽然目前多数研究仍认为*EGFR*突变NSCLC患者免疫检查点治疗效果不佳，但基于一些有效病例的出现，免疫检查点抑制剂对于*EGFR*突变患者的疗效，以及在可能的疗效预测标志物PD-L1的辅助下是否可以帮助更多患者受益等问题仍值得继续考量。

### 4.2 免疫检查点抑制剂联合TKI治疗的研究进展

TKI可诱导晚期*EGFR*突变NSCLC的快速应答，但通常在9个月-13个月发生获得性耐药现象^[3, 4]^。相比而言，免疫检查点抑制剂具有改善肿瘤患者免疫状态的特殊作用，可使疾病得到持续控制。如前所述，*EGFR*突变患者可出现PD-L1表达下调、低TIL浸润等抑制性免疫微环境，对免疫检查点抑制剂应答率较低。一些研究表明TKI可调节肿瘤免疫微环境，优化机体抗肿瘤活性，从而增强患者PD-1/PD-L1抑制剂获益几率^[46-49]^。机制可能有：①TKI可增强主要组织相容性复合体（major histocompatibility complex, MHC）Ⅰ和Ⅱ的表达，提高免疫提呈作用^[47]^；②TKI可通过促使Foxp3降解来减弱Treg细胞的抑制功能^[50]^，体内研究^[48]^也验证了TKI可抑制肿瘤生长并降低肿瘤微环境中Treg细胞浸润；③TKI可增强细胞毒性T淋巴细胞（cytotoxic lymphocyte, CTL）介导的的抗肿瘤活性，减少T细胞凋亡，增加IFN-γ产生^[51, 52]^。因此，近期多项相关研究正不断开展，探索联合应用TKI和免疫检查点抑制剂的疗效及毒性情况。

Ib期TATTON研究^[53]^评估了奥希替尼联合durvalumab治疗*EGFR*突变NSCLC的疗效。该研究将患者分为TKI初治组（剂量扩增疗法）和耐药组（剂量爬坡疗法），结果显示，在21例已经TKI治疗的患者中，12例达PR，9例达疾病稳定（stable disease, SD）；在10例TKI初治患者中，8例达PR，2例达SD。Gettinger等^[54]^评估了纳武单抗联合厄洛替尼治疗*EGFR*突变、化疗初治的晚期NSCLC患者的疗效和安全性，同样将患者分为TKI初治组（1例）和治疗后耐药组（20例）。结果显示，患者ORR为19%（4/21），中位反应持续时间（median duration of response, DOR）未达到，24周PFS为51%，1年总生存率为73%。其中TKI初治的1例患者达PR，DOR大于72.3周；厄洛替尼耐药患者组的20例患者中，15%（3/20）达PR，45%（9/20）最佳疗效反应为SD。此外，不良反应（adverse events, AE）在可接受范围内，没有治疗相关肺炎出现，3级-4级治疗相关AE为23.8%（5/24），19%（4/21）的患者由于AE中止治疗。尽管该试验获得令人满意的效果，但考虑到随访期较短，纳入患者数较少，需要进一步随访并持续观察来评估这种联合治疗的真正效益。很多研究者仍不建议*EGFR*突变患者联合应用TKI靶向药物与免疫检查点抑制剂，认为其有效率远低于一线TKI靶向药物，并且有可能导致爆发进展。

此外，毒性作用的叠加是一个不容忽视的重要问题。一项研究评估了厄洛替尼联合阿特珠单抗（atezolizumab）的安全性^[55]^，结果显示最常见的3级-4级AE为发热和谷丙转氨酶（alanine transaminase, ALT）升高，该研究无肺炎和5级AE发生。而TATTON研究^[53]^显示联合奥希替尼与durvalumab用药时有38%（13/34）的患者出现间质性肺疾病[其发生频率远高于单药奥希替尼（2.9%）或durvalumab（2%）]，且15%（5/34）为3级-4级，59%患者由于AE而终止治疗。一项研究总结了2015年4月-2017年3月期间共20, 000例*EGFR*突变NSCLC患者的资料^[56]^，显示间质性肺疾病的发生率在全部人群中占4.8%；在5, 777例接受EGFR抑制剂的患者中占4.59%；在5, 178例接受PD-1抑制剂治疗的患者中占6.4%；然而在同时使用过EGFR抑制剂和PD-1抑制剂的患者中却高达25.7%，可见，两者联合使用发生间质性肺疾病的风险提高了5.09倍。因此该实验研究者认为，*EGFR*突变晚期NSCLC患者应尽量选择靶向药物、放化疗以及贝伐单抗等抗血管生成药物，PD-1抑制剂或者两药联合应用并不是其合适选择。

目前多项联合两药的临床试验正在进行，未来需要进一步解决的难题主要有：①两药安全性和有效性问题的深入探讨，以及如何区分免疫相关和TKI诱导的AE；②药物相关间质性肺疾病和肝毒性的发生无法避免，且发生人群无法预测，仍需开展大量实验探究有效的疗效和毒性预测生物标志物，为临床决策提供有价值信息。

## 5 总结与展望

近几年，针对*EGFR*突变靶向药物TKI在NSCLC治疗中虽取得一定成效，但多数患者在1年左右出现耐药，新型治疗方案的研发引起了人们的关注。然而，多项基础研究与临床研究结果相互矛盾，其单独或联合应用备受争议。我们的系列综述显示，*EGFR*突变基因可能造成PD-L1低表达、抑制性免疫微环境及低TMB等免疫特征，成为机体抗肿瘤免疫反应较弱的可能机制。未来的研究仍存在一些挑战。首先，PD-L1检测技术及免疫检查点抑制剂疗效预测标志物的研究尚不统一。针对*EGFR*突变NSCLC患者，ATLANTIC研究^[45]^提示PD-L1表达似乎可成为筛选适合免疫治疗的生物标志物，而表达量超过25%可能成为筛选阈值。但需进一步实验验证，并动态监测PD-L1表达随治疗发生的改变。另外，其他生物标志物，如TIL、T细胞库特征、肿瘤TMB和新抗原及机体整体状态在疗效预测方面都被认为具有一定潜力，建议多种标志物联合动态监测，最大限度地提高患者免疫治疗效果。其次，尽管很多研究证实*EGFR*突变诱导的免疫抑制微环境与多种因素有关，但我们无法估计这几个连续变量的确切界值及他们之间的相关性。最后，TKI与免疫抑制剂联合应用虽取得一定成效，但治疗相关AE的叠加却使部分实验提前终止，对毒性反应的提前预测至关重要。免疫治疗应用于*EGFR*突变NSCLC患者的证据尚不足，多数实验并不支持其单独或联合应用，未来仍需开展大量随机前瞻性研究，为耐药患者寻找生存希望。
